# Can the Mismatch Negativity Really Be Elicited by Abstract Linguistic Contrasts?

**DOI:** 10.1162/nol_a_00147

**Published:** 2024-09-11

**Authors:** Stephen Politzer-Ahles, Bernard A. J. Jap

**Affiliations:** Department of Linguistics, University of Kansas, Lawrence, Kansas, USA; Department of Chinese and Bilingual Studies, Hong Kong Polytechnic University, Kowloon, Hong Kong; Centre for Cognitive and Brain Science, University of Macau, Taipa, Macau

**Keywords:** abstract linguistic processing, auditory deviance detection, event-related potential (ERP), mismatch negativity (MMN)

## Abstract

The mismatch negativity (MMN) is an event-related potential component that reflects pre-attentive change detection in the brain. As an electrophysiological index of processing that responds to differences in incoming consecutive stimuli, the MMN can be elicited through, for example, the presentation of two different categories of sounds in an oddball paradigm where sounds from the “standard” category occur frequently and sounds from the “deviant” category occur rarely. The specificity of what can elicit the MMN is yet to be fully defined. Here we test whether the MMN can be generated by an abstract linguistic contrast with no reliable acoustic cue. Previous studies have shown that the way in which an acoustic cue is used to elicit MMN is influenced by linguistic knowledge, but have not shown that a nonacoustic, abstract linguistic contrast can itself elicit MMN. In this study, we test the strongest interpretation of the claim that the MMN can be generated through a purely linguistic contrast by contrasting tenses in ablauting irregular English verbs (where there is no reliable acoustic cue for tense). We find that this contrast elicits a negativity, as do other linguistic contrasts previously shown to elicit MMN (a contrast between phonologically voiced and phonologically voiceless segments and a purely acoustic contrast between aspirated and unaspirated segments). The findings provide evidence that the MMN is indeed sensitive to purely abstract linguistic categories.

## INTRODUCTION

The human brain performs many kinds of processing on stimuli that it is not consciously paying attention to, or even consciously aware of. One way we know this is from the *mismatch negativity* (MMN), which reflects the detection of changes in incoming stimuli even if the stimuli are not attended to (Näätänen et al., 2007). A common way to elicit the MMN is to present stimuli to a person in an oddball paradigm while the person pays attention to something else. In an oddball paradigm, two kinds of stimuli are repeated many times, but one kind of stimuli is repeated frequently and the other is repeated rarely (e.g., *da da da da da da **pa** da da da da **pa** da da da da da da da da **pa** da da da* …; in MMN research the frequently repeated class of stimuli are called “standards,” and the rarely repeated are called “deviants”). A stimulus elicits a more negative-going event-related potential (ERP) when it is presented rarely (i.e., as a deviant) than when it is presented frequently (i.e., as a standard); this difference is the MMN. The generation of the MMN is generally described as resulting from the process of noticing that an incoming stimulus does not match with the memory representation of recently presented stimuli (Näätänen et al., 2007), or the process of updating a predictive model when encountering a stimulus that doesn’t match what was expected (Bornkessel-Schlesewsky & Schlesewsky, 2019; Winkler, 2007). The MMN is one of the best understood and most widely used components in ERP research; it has been used as an instrument to test theories of language processing and representation (e.g., Eulitz & Lahiri, 2004), to probe language acquisition outcomes that may not be apparent in behavior (e.g., Lu et al., 2015), to examine differences between people with and without autism (e.g., O’Connor, 2012), and to help detect various neuropsychological conditions (e.g., Schall, 2016; Zarza et al., 2007), as well as many other basic and clinical applications (for review see Näätänen et al., 2007).

The MMN is not simply a detector of any differences, however. One of the reasons the MMN is so useful is that its sensitivity to differences is informed by human psychology. This has been demonstrated by, for example, Phillips, Pellathy, Marantz, and colleagues (2000), who showed that human listeners only show an MMN response when a series of sounds they hear falls into an oddball paradigm based on the phonological categories of their language. If English speakers hear a series of sounds with varying amounts of aspiration, such that 1/8 of the sounds would be perceived as “da” and 7/8 of the sounds perceived as “ta,” they would have an MMN response. On the other hand, if they hear a series of sounds with an identical amount of variation in aspiration but with the mean amount of aspiration shifted up such that half of the sounds were perceived as “da” and half as “ta,” then they no longer have an MMN response, because this series of sounds does not form an oddball paradigm. An even more powerful demonstration of the reliance of the MMN on language-specific phonological categories is provided by Kazanina and colleagues (2006), who tested a similar aspiration contrast on both Russian speakers, for whom this contrast is meaningful in their language, and Korean speakers, for whom it is not. For Russian speakers, [t] and [d] belong to different phonemes (/t/ and /d/), just like for English speakers in the study described above; for Korean speakers, however, [t] and [d] in syllable onsets are realizations of the same phoneme, which is pronounced as [t] word-initially but as [d] intervocalically. Russian speakers, but not Korean speakers, showed an MMN for the contrast between [t] and [d] in this study, using a paradigm comparable to the one by Phillips, Pellathy, Marantz, and colleagues (2000).

Findings like these demonstrate that the process by which the brain detects changes, as indexed by the MMN, uses knowledge of the listener’s language system. Furthermore, the brain performs a substantial amount of processing on incoming stimuli—including placing these stimuli into categories, using those stimuli to make predictions about upcoming stimuli, and updating its predictive model when these predictions turn out to be wrong (Bornkessel-Schlesewsky & Schlesewsky, 2019; Winkler, 2007)—that the listener is not even consciously paying attention to.

Based on findings like these and others that will be discussed below, it is often claimed that the MMN is sensitive to, or can be elicited by, abstract linguistic features or rules. While this could mean several different things, we believe no study yet has convincingly demonstrated that the MMN can truly be elicited by contrasts in abstract linguistic features—at least not under the strongest possible interpretation of this claim. The present study will test the strongest version of this claim by attempting to elicit MMN in stimuli in which there is no physical cue whatsoever to the crucial contrast. First, however, we must establish what exactly is meant by a claim that the MMN is sensitive to abstract linguistic contrasts, and why previous studies have not yet proven that.

### The MMN and Abstract Linguistic Contrasts

There are different ways in which linguistic knowledge could matter to the MMN. In particular, sensitivity to a linguistic contrast that is cued by a physical contrast is not the same thing as elicitation by a linguistic contrast itself. Some instances of MMN are certainly dependent on linguistic knowledge and contrasts, because the MMN relies on these in order to put incoming stimuli into categories which then fall into an oddball relationship and elicit MMN, as described above. That does not mean, however, that the linguistic contrast or features themselves elicit the MMN; rather, the MMN itself is still elicited by a physical difference, just one in which the relevant boundary has been determined by linguistic knowledge. For example, in the Phillips, Pellathy, Marantz, et al. (2000) study on aspirated versus unaspirated categories, the MMN is elicited by a difference between long and short voice onset times; the brain just uses linguistic knowledge to decide where to draw the line between “short” and “long.” The same can be said of Kazanina et al. (2006)—while the cross-language comparison in that study provides compelling evidence that linguistic knowledge is used to categorize (or predict) incoming sounds and that this knowledge allows the sounds to be categorized in a way that then generates an MMN, it does not prove that the linguistic contrast itself (as opposed to the physical, acoustic information that is then categorized according to linguistic knowledge) is what elicits the MMN. One could call this an MMN elicited by a physical difference that is informed by linguistic knowledge, as opposed to an MMN elicited by a purely linguistic difference.

A related line of research has argued that the MMN is sensitive to linguistic features by demonstrating that the MMN can be elicited in situations where stimuli differ in multiple features but only one of those features yields an oddball grouping. For example, Phillips, Pellathy, and Marantz (2000) found MMN responses when English-speaking participants heard series of sounds like *ba ba ga da ba ga da da **ta** ga da ga ba da da ga **ka** ba ga **pa***. In a stream like this, there is no unique sound that occurs much more frequently than the others, and thus no particular sound can be considered a standard; however, if we ignore place of articulation, we can see that most of the sounds are unaspirated (*ba*, *da*, and *ga*) and only a few are aspirated (*pa*, *ta*, and *ka*), and thus the class of unaspirated sounds works as a standard and aspirated sounds work as the deviant. Similarly, Fu and Monahan (2021) elicited MMNs by contrasting retroflex versus nonretroflex sounds while varying other features (i.e., retroflex [ʂ, tʂ, tʂ^h^, ɻ] vs. nonretroflex [s, ts, ts^h^, l]). Findings like these have been taken to mean that the MMN can be elicited by a contrast between linguistic features. A similar effect has been found with lexical tones; e.g., Wang et al. (2012) and Politzer-Ahles et al. (2016) found MMNs in Mandarin-speaking participants hearing series like *ú í é é **è** ú ú á ú í é á **à** é í é **ù***, where the vowels themselves are not in a standard–deviant relationship but the tones are (the diacritics represent tone categories). Another example of this paradigm comes from Schluter et al. (2017), who found MMNs when voiced fricative deviants ([v], [z], and [ʐ]) were occasionally interspersed among voiceless fricative standards ([f], [s], and [ȿ]) even though the place of articulation varied, directly paralleling the aspirated-deviant/unaspirated-standard sort of paradigm of Phillips, Pellathy, and Marantz (2000) described above.

Studies like these, however, cannot in and of themselves be taken as proof that the MMN can be elicited by contrasts of abstract linguistic features. In fact, in the studies described in the previous paragraph, a single acoustic feature could yield the necessary deviant/standard oddball ratio necessary to produce MMNs; no linguistic knowledge is required. For the study comparing voiced and voiceless fricatives, the presence of fundamental frequency in the voiced fricatives and its absence in voiceless fricatives is enough to recognize that they fall into separate categories. There are also fairly stable spectral differences between the retroflex and the nonretroflex sounds described above. Likewise, for the studies on Mandarin tone, all the brain needs to do is low-pass filter the input, discarding information other than the fundamental frequency, in order to recognize that the stimuli fall into two discrete categories. In fact it is already known that the brain can keep track of deviant/standard ratios of many physical features simultaneously, even in nonlinguistic stimuli (see, e.g., Pakarinen and colleagues, 2010, who obtained MMNs even when they simultaneously varied seven separate features of nonlanguage stimuli [loudness, duration, pitch, etc.] such that every stimulus was simultaneously a deviant for certain features and a standard for others), and thus it is quite plausible that the brain would automatically track all these sorts of acoustic information at once in linguistic stimuli, without necessarily needing to make reference to linguistic knowledge. Thus, these types of studies are actually much weaker evidence for MMN sensitivity to abstract linguistic features than studies like Phillips, Pellathy, Marantz, et al. (2000) and Kazanina et al. (2006).

The study by Phillips, Pellathy, and Marantz (2000) is somewhat more complicated, because the voice onset times of the stimuli varied in such a way that linguistic knowledge is indeed necessary to categorize the stimuli into standard and deviant groups (for the same reason as in Phillips, Pellathy, Marantz, et al., 2000, described in the previous section). Furthermore, the relevant boundary between aspirated and unaspirated categories differed between places of articulation (for example, since velar stops tend to have longer voice onset times [VOTs] than alveolar and labial stops, a given VOT that is interpreted as “aspirated” in a labial context might be interpreted as “unaspirated” in a velar context), so an MMN could not be generated in this study by simply discarding the place of articulation information. Nevertheless, this could be viewed as an example of an MMN elicited by a physical contrast that is informed by linguistic knowledge (albeit in a more complicated and multidimensional way than, e.g., the Mandarin tone studies), rather than an MMN elicited by a purely linguistic contrast, for the same reasons described above in reference to Phillips, Pellathy, Marantz, et al. (2000) and Kazanina et al. (2006).

Here, when we ask if the MMN can be elicited by *abstract* linguistic contrasts, we mean ones that cannot be detected based on physical cues alone. Traditional accounts of the MMN, in which the MMN is generated by a mismatch between an incoming stimulus and a memory representation of preceding or predicted stimuli (Bornkessel-Schlesewsky & Schlesewsky, 2019; Näätänen et al., 2007; Winkler, 2007) arguably could predict MMN to be elicited by contrasts without physical cues, since other features could also result in a mismatch between incoming and previous representations. As discussed above, however, most studies on language-elicited MMNs have not demonstrated this, as they rely on contrasts that have physical cues—although a few studies, such as Phillips, Pellathy, and Marantz (2000), Phillips, Pellathy, Marantz, et al. (2000) and Kazanina et al. (2006), do indeed meet this criterion because the standard–deviant contrast cannot be noticed based on physical cues alone but only on physical cues interpreted via linguistic knowledge.

Furthermore, when we ask if the MMN can be *elicited by* abstract linguistic contrasts, we mean whether the contrast itself, without being realized on any discrete physical cue, is sufficient to generate the MMN. Studies like Kazanina et al. (2006), while they do convincingly demonstrate that linguistic knowledge is necessarily involved in the MMN, do not demonstrate that linguistic contrasts alone can elicit MMN, because ultimately the MMN in these studies is elicited by a physical cue that has been perceived through the lens of linguistic knowledge.

We believe most studies commonly cited as evidence for the sensitivity of the MMN to abstract linguistic contrasts do not actually provide evidence for this strongest possible conception of what it would mean for the MMN to be *elicited by abstract linguistic contrasts*. It would be valuable to figure out whether the MMN can really be elicited by abstract linguistic contrasts in the way sketched above. If the MMN can indeed be elicited by a linguistic contrast with no physical correlate, that would provide the strongest yet demonstration of what kinds of representations the MMN operates on. On the other hand, if the MMN cannot be elicited by such an abstract linguistic contrast, this would not undermine the more nuanced conclusions of the previous studies (e.g., Kazanina et al., 2006, would still provide convincing evidence that linguistic knowledge is involved in the process that generates the MMN, even if only as a filter for incoming acoustic information), but it would set important boundary conditions on what kinds of information can generate MMNs and thus would provide an important refinement of our current understanding of how the MMN is generated.

### Other Studies Reporting Modulation of MMN by Abstract Linguistic Information

Having sketched out a picture of what it would mean for the MMN to be elicited by an abstract linguistic contrast, we should review a few other lines of research that show sensitivity of MMN to linguistic information.

Particularly relevant is literature showing that the amplitude of the MMN is sensitive to phonological underspecification in the standard. Eulitz and Lahiri (2004) showed that the MMN is smaller when the key feature distinguishing the standard and deviant is underspecified in the standard—for example, when the deviant is a dorsal vowel [o] and the standard is a vowel [e] which, while pronounced with coronal place of articulation, putatively has no place of articulation specified in its underlying phonological representation. Many subsequent studies have replicated this pattern with other features and in other contexts. The argument is that when people hear a standard, they convert it into a memory representation and the MMN is ultimately elicited when an incoming deviant mismatches with that memory representation, but when the standard is underspecified for some feature then there is no feature in the memory representation for the deviant to mismatch with, and thus not much MMN is elicited. While a modulation of MMN amplitude like this could emerge for nonlinguistic reasons as well (for review see Politzer-Ahles et al., 2016), subsequent research has likely ruled out many of these reasons. For example, Hestvik and Durvasula (2016) found that this underspecification effect emerges in a paradigm that encourages access to underlying phonological representations but not in a paradigm that only encourages access to surface phonetic forms; Politzer-Ahles et al. (2016) and Hestvik et al. (2020) found that this effect is dependent on the language background of the listeners and does not emerge in listeners who do not have the necessary phonological knowledge; and Meng et al. (2021) found that this amplitude modulation can even be triggered by phonological features spread from neighboring segments, in a way that is not easily explained without reference to features.

If these effects can only be explained by phonological underspecification and cannot be explained by any other factor, then these studies are strong evidence that abstract linguistic knowledge plays an important role in the generation of the MMN. However, they are not evidence that the MMN can itself be generated by a purely linguistic contrast. They are, like Kazanina et al. (2006), evidence that the MMN can be modulated by a physical contrast supplemented by, or fed through, linguistic knowledge.

Similarly, Vera-Constán and Sebastián-Gallés (2008) found larger MMN when a peripheral vowel is used as a deviant among more central standards, as opposed to when a more central vowel is used as a deviant among more peripheral standards. (A more recent and more detailed report by Polka et al., 2021, however, did not observe this effect in a similar manipulation.) This can be explained through the natural referent vowel framework (Polka & Bohn, 2003, 2011), which was developed to explain this and other perceptual asymmetries between central and peripheral vowels. Like the studies discussed above, though, this is evidence that the size of the MMN elicited by a physical contrast is modulated by linguistic knowledge, rather than that the MMN is elicited by a purely linguistic contrast itself. (It is not even necessarily evidence for that, because this effect may be due to the fact that peripheral vowels have more focal concentrations of acoustic energy, rather than to their linguistic status [Masapollo et al., 2017; Schwartz et al., 2005].) Another line of work has found that a larger MMN is elicited when the standards are prototypical realizations of a vowel in the listener’s language than when they are not (Shinohara et al., 2022). While this has important implications for the nature of the memory representation of the standard, the elicitation of the MMN itself is still based on a reliable physical difference between the deviant and the standard.

Several studies, mostly with nonlinguistic stimuli, have found that the MMN can be elicited by a deviant that does not fit a rule, as opposed to a deviant that does not fit a category. For example, when a sequence of standards is continually falling in tone from one standard to the next, but then the deviant has a higher tone than the preceding standard, MMN is elicited (Tervaniemi et al., 1994). This also works for multifeature rules; for example, in Houlihan and Stelmack (2012), standards had frequency and intensity varying in direct proportion whereas deviants had frequency and intensity varying in inverse proportion. Omission of an expected stimulus (e.g., Bendixen et al., 2009; Wacongne et al., 2011) is also a way of violating a rule, and also elicits MMN. However, these types of studies are still not evidence that the MMN can be elicited by an abstract contrast or rule; in these studies, the violation is still cued by discrete physical/acoustic information (i.e., a mismatch between the acoustic features of the incoming stimulus [or lack thereof] and the acoustic features of the stimulus that would be expected based on the preceding rule). While many of these studies are discussed as examples of MMNs related to “abstract” rules, the use of the term “abstract” in these studies is not referring to the same kind of “abstractness” we have outlined above.

Numerous studies have observed MMN for contrasts between syntactically correct phrases and phrases with syntactic or morphosyntactic grammatical errors (Hasting et al., 2007; Herrmann et al., 2009; Pulvermüller & Assadollahi, 2007; Pulvermüller et al., 2008; Shtyrov et al., 2003). However, while these might at first glance seem like evidence for elicitation of MMN by an abstract linguistic contrast, they actually are not, because they also rely on a unique acoustic correlate that cues the syntactic contrast—for example, comparing the German grammatical phrase *ein Falter* (“a butterfly”) with the German ungrammatical phrase *ein faltet* (*“a folds”), where the difference is uniquely cued by the final consonant and thus the brain response may reflect detection of acoustic change rather than detection of abstract category change. Other studies on MMN elicitation through syntax investigate how certain conditions generate larger MMN amplitude (Hanna et al., 2017; Kubota et al., 2020): In these studies, the MMN is elicited by an obvious physical contrast and that physically elicited MMN just happens to be larger when it occurs on a deviant that is ungrammatical. For example, in Hanna et al. (2017), deviants had an extra particle that standards did not. (Standards were German phrases like *sie fügen es* [“they joined it”] and deviants were phrases like *sie fügen es an* [“they joined it together”].) The contrast that elicited the MMN was always a contrast between phrases without an extra one-syllable particle and phrases with it; the “syntactic” aspect of the MMN they observed is the fact that this MMN is larger when the deviant is an ungrammatical phrase than when the deviant is a grammatical one. Thus, “syntactic MMN” studies—at least studies conducted in the way the studies thus far have been—are not evidence that the MMN can itself be triggered by an abstract linguistic contrast, only that it may be modulated by abstract linguistic properties given that it has already been triggered by some lower-level contrast. A similar observation can be made about studies showing that word deviants elicit larger MMNs than nonword deviants (e.g., with English speakers, *bipe *bipe *bipe **bite** yields a larger MMN than pipe pipe pipe *pite; see Politzer-Ahles & Im, 2020, for review): In these studies, the MMN itself is elicited by a low-level acoustic contrast (the difference between [t] and [p]) rather than by a contrast in lexicality.

Hu and colleagues (2020) presented participants with oddball sequences of characters in which deviants represented words belonging to different semantic categories than standards (e.g., action words vs. color words), and they claim to have observed a visual MMN for this contrast (although this finding depends on a subjective choice of analysis window). Their study, however, was meant to examine the extraction of semantic information from radicals (orthographic components of Chinese characters), and thus they used stimuli in which the semantic contrast was also supported by the presence of a physical cue in the form of a radical; in fact, in one of their experiments that used stimuli without radicals to cue the semantic contrast, they did not observe an MMN. This study, therefore, arguably does not provide support for an MMN based on a completely abstract linguistic contrast.

Finally, perhaps the best demonstration yet for an MMN elicited by a bona fide linguistic contrast is a study by Monahan and colleagues (2022), who presented English-speaking listeners with standards and deviants that were distinguished by a phonological cue which has variable acoustic realization. Specifically, the difference between standards and deviants was that one set was phonologically voiced (e.g., /ba, da, ga, va, za/) and one was phonologically voiceless (e.g., /pa, ta, ka, fa, sa/). In American English, the distinction between voiced and voiceless stops (/b, d, g/ vs. /p, t, k/) is typically realized mainly by duration of aspiration (“voiced” /b/ is actually usually voiceless and unaspirated in word-initial contexts). On the other hand, the distinction between voiced and voiceless fricatives (/v, z/ vs. /f, s/) is realized by vocal fold vibration. Nevertheless, these fit into phonological categories: Voiceless unaspirated stops undergo many of the same phonological processes as truly voiced fricatives, and voiceless aspirated stops undergo many of the same phonological processes as voiceless fricatives. Therefore, in this study, the standards and deviants were distinguished by an abstract phonological feature but not by any unique acoustic cue. An MMN was elicited in this situation, which suggests that MMN can indeed be elicited by abstract phonological contexts. (Phillips, Pellathy, & Marantz, 2000, which used {[b], [d], [g]} standards and {[p], [t], [k]} deviants, also has a similar logic, because the categorical boundary between “aspirated” and “unaspirated” stops lies at different voice onset times for stops with different places of articulation, and therefore the linguistically based boundary used to categorize sounds as standards and deviants is not the same across all the tokens. Monahan et al., 2022, however, goes even a step beyond this, by using a contrast in which the physical correlate is qualitatively different, rather than quantitatively different, across different tokens.) Monahan et al. (2022) is probably the strongest evidence yet for an MMN elicited by a linguistic contrast. Even in this study, however, the linguistic contrast is realized through physical cues; whereas previous studies used a single physical cue to trigger a linguistic contrast, this study used two cues, such that the brain would need to check two different acoustic features against its memory representation or predictive model rather than just checking one—and the determination of which cue to use is based on a reliable rule (i.e., if the sound is a fricative, look at voicing, and if the sound is a stop, look at aspiration).

### The Present Study

As reviewed above, while there is substantial evidence that linguistic knowledge can modulate the MMN and that the MMN can be elicited by physical contrasts in situations that depend on linguistic knowledge to make the contrast work, there is very little extant evidence that the MMN can be directly elicited by an abstract, purely linguistic contrast. The strongest evidence for such an effect would be an MMN elicited in a situation where deviants and standards fall into different linguistic categories and that difference is not at all cued by any reliable physical correlate. If we can observe an MMN in a situation like that, it would expand the boundaries of what kinds of information and representations can be processed pre-attentively by the brain and would advance our understanding of what exactly the MMN is doing (because, while current accounts of the MMN generation process arguably might predict an MMN to be possible in this sort of situation, very few studies have actually observed such an MMN). On the other hand, if we fail to observe an MMN in that kind of situation, that would help refine our understanding of the MMN by helping to establish boundary conditions on what kind of information and what kind of representations are processed by the functions that generate the MMN. If an MMN is absent in our experiment, it may suggest that linguistically meaningful differences require the accompaniment of physical contrasts, as in the previously discussed studies, to elicit an MMN.

In the present study we test this by seeing if an MMN can be generated by a morphosyntactic contrast with no reliable physical correlate: the distinction between past- and present-tense ablauting irregular English verbs. We present participants with oddball sequences of verbs in which a small percentage of the items are past-tense verbs (*gave*, *met*, and *sank*) and a large percentage are present-tense verbs (*pave*, *get*, *thank*, and several other present-tense filler verbs), and with the converse (sequences with a small percentage of present-tense verbs *pave*, *get*, and *thank*, and a large percentage of past-tense verbs *gave*, *met*, *sank*, and other filler past-tense verbs). In these sequences, the words form a deviant/standard oddball paradigm in terms of tense, but not in terms of any acoustic feature. This would thus represent the strongest possible test to date of an MMN elicited by a purely linguistic contrast with no cueing from physical/acoustic information. (Note that a failure for this past–present contrast to elicit MMNs would not rule out the possibility that some other abstract contrast might elicit MMNs. It would, however, challenge the assumption that the MMN can be generated by abstract contrasts, in a way we do not believe this assumption has been challenged before. Likewise, finding that one abstract contrast can elicit MMNs would not entail that all abstract contrasts can do so, but it would nevertheless show the strongest evidence yet that abstract contrasts can in principle elicit MMNs.)

(Previous investigations of irregular past tense forms in English using magnetoencephalography (Stockall & Marantz, 2006) have found that both regulars and irregulars are composed from the stems. This observation is contrary to the dual mechanism model (Pinker & Prince, 1988), which proposed that only regular past tense verbs are generated by rule from stems while irregulars are stored as separate entries in the lexicon. The Stockall and Marantz (2006) single-mechanism account is also distinct from the connectionist account (Rumelhart & McClelland, 1986), which claims that morphological similarity forms the network between all past tense forms and the stems. Fruchter et al. (2013) further supported this single-mechanism model by observing a decomposition response from irregular verb forms into stems and affixes before lexical processing in a masked priming experiment. Under this account, we would expect the irregular past tense verbs to be processed similarly to regulars, and while there may potentially be asymmetry of MMNs due to underspecification of present tense features, it would not influence our predictions on the manipulation being a purely abstract contrast.)

One previous study has tested something very similar to this. Muller-Gass and colleagues (2007) examined a contrast between 34 words and 34 pseudowords, such that there was no invariant acoustic cue to signal the contrast. Participants performed an active task in their study, but it was a task related to a different aspect of the stimuli (judging the pitch of the stimuli, which was unrelated to the lexicality of the stimuli), so their attention was not drawn to the lexicality contrast. There are, however, a few limitations to this study that motivate the need for a new study. The syllables in that study were all CVC.CVC disyllables (e.g., German *Zirkus* [circus] vs. *Zirtel) and the report does not indicate the recognition points of the words or the points at which the pseudowords are recognized to be pseudowords; thus, it is possible that potential ERP effects are obscured by variation in timing (ERPs were time-locked to the onset of the second syllable). This study did observe small numerical (although apparently not very statistically robust) differences between the ERPs for standards and deviants, but this difference does not appear to have been an MMN effect: Deviants yielded slightly more positive ERPs than standards about 200 ms after the onset of the second syllable, and slightly more negative about 400 ms after. The study’s sample size (*N* = 14), while typical for studies at the time this study was conducted, is small by current standards, and likewise the statistical analysis upon which the conclusions are based does not hold up to current standards (e.g., different amplitude measures are used in different time windows for no clear reason, and amplitudes are measured around peaks in the waveforms of individual conditions even though these kinds of peaks are not special [Luck, 2014]). Therefore, while this study provides some suggestive evidence that the brain may indeed be capable of rapidly detecting abstract linguistic differences, the conclusions that can be drawn from it are not clear, so further evidence is needed.

Another study somewhat similar to ours is that by Kotchoubey and Lang (2001), who were able to elicit a P300 for a semantic class distinction (animal words vs. words from other semantic classes) without an invariant acoustic correlate. This, however, was in an active oddball task in which participants were performing a task that required them to pay attention to animal words, and thus it does not tell us whether the pre-attentive MMN will also be sensitive to abstract contrasts like this.

In addition to the abovementioned contrast between past- and present-tense verbs, the present study will also include two manipulation checks: one with an abstract, but still phonological, contrast, and one with a simple acoustic contrast.

As a relatively abstract manipulation check, the study will also include a replication of Monahan and colleagues’ (2022) design that observed MMN for a phonological contrast that is realized by different acoustic cues on different segments. Participants will hear stimuli in oddball sequences such that some of the stimuli start with phonologically voiced consonants (/*ba*, *da*, *ga*, *va*, *za*/) and some start with phonologically voiceless consonants (/*pa*, *ta*, *ka*, *fa*, *sa*/); in one block the voiced condition will be the standards, and in another block the voiceless will be the standards. Crucially, the specific acoustic cues for voice are different in stops ({*ba*, *da*, *ga*} vs. {*pa*, *ta*, *ka*}) than they are in fricatives ({*va*, *za*} vs. {*fa*, *sa*}): The voicing distinction in English onset stops is mainly cued by voice onset time, whereas the voicing distinction in English fricatives is mainly cued by the presence or absence of F0 within the fricative. Following Monahan et al. (2022), we expect to see an MMN elicited for the voice contrast here. We include this manipulation because if we fail to elicit an MMN in the tense contrast described above, it will be important to demonstrate an MMN elsewhere in the experiment to prove that the experiment is capable of finding MMNs, and we want this manipulation check to be as abstract as possible to be maximally comparable to the critical tense conditions (a manipulation check consisting of, e.g., pure tones with an obvious pitch difference would not be appropriate for this purpose, because these would elicit a far larger MMN and would not demonstrate that our setup is sufficiently sensitive to detect MMNs elicited from a more subtle, comparable contrast).

On the other hand, the simple acoustic manipulation check will be a VOT distinction that is cued by a single consistent acoustic feature across all the standards and deviants, that is, just *ba* vs. *pa*. There will be intercategory variation (we will use multiple natural tokens of each stimulus), but the acoustic cue that distinguishes standards and deviants is always VOT. This is included to ensure that we are capable of eliciting MMNs with a contrast that has reliably generated MMNs previously; if neither the critical manipulation nor the more abstract manipulation check above elicits MMNs, it would be necessary to have MMNs in this simpler manipulation check in order to conclude that those conditions really do not elicit MMN or elicit MMNs too small to detect in our paradigm (as opposed to concluding, e.g., there was a flaw in our experiment).

If abstract contrasts are capable of generating MMNs, what exactly is the mechanism by which that could occur in the present study? In typical linguistic MMN paradigms, repeated presentation of the standard allows listeners to generate a strong prediction for incoming sensory input. (This prediction might be an accurate representation of the exact physical stimulus, in the case of paradigms that use single tokens, or at least a relatively strict range of possible physical stimuli, in the case of paradigms that use some within-category variability.) Thus generated, these fairly specific predictions minimize the degree of mismatch between the physical stimuli and the listener’s internal predictive model, so little to no MMN is elicited when hearing standards. Then, when the listener hears a physical sound that deviates from this prediction, an error signal is generated, and this is reflected by increased MMN (Bornkessel-Schlesewsky & Schlesewsky, 2019; Winkler, 2007). It is not clear that the present paradigm could generate MMNs in this way, since the standards do not share any physical features that would allow for the generation of these sorts of specific predictions. That is to say, even if the listener has successfully abstracted some kind of “past-tense” feature from the standards, they will still have no prediction for, for example, what will be the first sound in the incoming stimulus.

Nevertheless, listeners may still be able to use the previously extracted abstract feature to constrain predictions in a way that could yield an MMN, albeit perhaps on a longer timescale than is usually observed in MMN studies. Encountering the initial speech sound of some word sets off a recognition process in which the listener activates cohorts of candidate words—for example, all words that start with this sound—and then narrows down the cohort as further information arrives. There is substantial evidence (e.g., Andrews & Bond, 2009; Shuai & Gong, 2014) that top-down information is also used to help select or constrain candidates (although there is debate over precisely how this is implemented). In the present study, then, participants may use the predictive model generated on the basis of the standards to constrain the cohort of words they activate to, for example, only past-tense verb forms that start with the sound they have just heard. In that case, an MMN could be elicited when enough bottom-up input has been heard to eliminate all candidates, at which time the processor must recognize that a prediction error has occurred.

The account sketched above is based entirely on forward predictions. The generation of the MMN process may also involve some backward-looking comparisons, though; in other words, the MMN might not be based only on encountering input that does not match a forward prediction, but may also be influenced by some process that takes new input and actively compares it against features extracted from the standards. The clearest example of this comes from MMN research on phonologically underspecified features (e.g., Eulitz & Lahiri, 2004, among others). A central argument of this literature is that a feature that is underspecified from the standards is not incorporated into the predictive model at all: For example, if standards are all coronal, and the Coronal feature is underspecified, then there is no forward prediction for incoming sounds to be coronal. Instead, if an MMN is elicited by incoming noncoronal stimuli, this occurs when they are compared against the mental model of the standards and are found not to mismatch but also not to match. In other words, these MMNs could be explained without reference to forward prediction of specific sounds or features and instead be explained as resulting from a comparison process which presumably happens later. Likewise, in the present study, it may be possible that listeners retrieve some tense feature from incoming deviants (presumably sometime after the uniqueness point of the stimulus is heard) and then check that against their existing predictive model and notice that it does not match.

We note that the abovementioned findings on underspecification could be accounted for just by forward prediction, and without assuming any sort of backward-looking comparison process, as long as we assume that the low-level acoustic difference between standards and deviants also contributed to the MMN. Under that sort of account, the asymmetry arises because blocks with specified standards have two contributors to the standard–deviant mismatch (the forward prediction about the specific stimulus acoustics turns out to be incorrect, and the forward prediction about phonological features turns out to be correct), whereas blocks with underspecified standards only have one contributor to this mismatch (the forward prediction about specific stimulus acoustics turns out to be wrong, but there is no prediction about phonological features). An account like this might predict that, with sufficient acoustic variability in the stimuli, there would be no MMN at all when the standard is phonologically underspecified for the critical feature, and that is precisely what sometimes happens (e.g., Politzer-Ahles et al., 2016). Thus, there may not be much evidence that the MMN involves anything other than forward prediction, and thus there may not be much evidence for the second account we have sketched here about how an MMN might arise after the listener recognizes the tense feature of the deviant.

Under either of these accounts, one might expect that the MMN for abstract contrasts would emerge relatively late (although prediction errors related to abstract category distinctions have yielded very early sensory responses in some studies, such as that by Dikker and colleagues, 2009). We believe such an effect could still be considered an MMN, because we are presupposing a functional definition rather than a temporal definition of ERP components—for example, if the P600 can be a late P3 (Bornkessel-Schlesewsky et al., 2011; Sassenhagen et al., 2014), we believe a fairly late negativity can still be an MMN if it is elicited in roughly the same way as other MMNs are elicited (i.e., a nonattentional oddball paradigm).

## MATERIALS AND METHODS

Stimuli and experiment presentation code, anonymized data, and analysis code for this study are available at https://osf.io/yx9zm/. This study was carried out as a registered report; the Stage 1 protocol for the study was reviewed before data collection and is archived at https://osf.io/wq43b.

### Participants

Data were recorded from 71 adult native speakers of English. Of these, data from one participant were excluded from the analysis because the participant dropped out of the experiment early; data from one more were partially lost due to experimenter error; and data from nine more were excluded because of low trial counts remaining after artifact rejection. This left data from 60 participants (28 men and 32 women, mean age 34, range 18–50) who had at least 30 trials in every condition. Participants were mostly right-handed (four were left-handed and one ambidextrous) and had no hearing impairment, history of neurological or psychiatric diagnoses or injury, recent (within 6 months) use of psychoactive medication, or exposure to other languages before age 5.

Setting a minimum trial count of 30 trials per condition as the inclusion criterion for participants is a deviation from our pre-registered plan; our original criteria were at least 50 trials per condition, a signal-to-noise ratio significantly above 3 dB (based on the method by Parks et al., 2016), and no more than three bad channels. During data collection, however, we found that this set of criteria was very conservative—only 16 of our participants met this inclusion threshold—and was more strict than comparable studies. Therefore, we chose to relax the criteria; we report an exploratory analysis of the smaller sample meeting the original conservative inclusion criteria in the Supporting Information, available at https://doi.org/10.1162/nol_a_00147.

The sample size of 60 participants was determined by a simulation-based power analysis for a difference between two paired conditions (i.e., one independent variable with two levels). We assumed an effect size of *d* = −0.37, which was the effect size (mean divided by standard deviation) of the MMN wave at Cz over 200–400 ms from Politzer-Ahles et al. (2016). That condition elicited one of the weakest MMNs in that study (mean = −0.84, *SD* across 24 participants = 2.28) and thus is a safe comparison for this study, where we may also need to be able to detect very weak MMNs. We simulated datasets with this effect size and with various numbers of participants and deviants (and with other variance components—e.g., variance across trials within a participant—taken from the MMNs in Schluter et al., 2016; this was done because we had those values readily available from that study and not from Politzer-Ahles et al., 2016, and in our previous experience, these particular variance components have not differed much from study to study) and a standard:deviant ratio of 85:15 in each simulation. Power was the percentage of simulations (for each sample size) for which the deviant condition was significantly more negative than the standard condition in a one-tailed *t* test (*α* = 0.05). The analysis showed that increasing the number of deviants beyond about 100 does little to increase power, but increasing the number of participants helps. We chose 60 participants and 120 deviants presented per condition as an optimally feasible combination to get 80% power.

Lowering the number of trials per condition necessary to include a participant in the analysis, however, increases the variance of the effect, which may decrease power. We re-ran the power analysis described above (but this time only for a sample size of 60 participants) with a lower number of trials, to account for the fact that some participants had a lower number of trials than what our original power analysis assumed. This analysis yielded 76% power, which we considered a negligible reduction compared to the power of the originally registered plan.

Participants provided informed consent (application #HSEARS20180409003) and were paid 300 HKD for the entire experiment session. Procedures were approved by the institutional review board of the Research and Innovation Office at the Hong Kong Polytechnic University.

### Materials

The list of words used in the experiment is shown in Table 1. For the critical blocks with a tense contrast, we chose three unambiguous irregular past-tense verbs and three unambiguous irregular present-tense verbs to serve as standards and deviants; the words used as deviants in one block were used as standards in another block. The past- and present-tense verbs differ only in the initial consonant, and as far as we can tell there is no systematic phonetic or phonological difference between these two sets. In order to make sure participants can recognize these as past- or present-tense verbs, we avoided any words that are homophonous with other words (e.g., we did not use *won* as a past-tense verb because its spoken form sounds the same as *one*, which is not a past-tense verb) or words which are commonly used as deverbal nouns (e.g., *run* is often used as a verb [“I have to run”] but also often used as a noun [“I went for a run”]).

**Table 1.** Word list for experiment

Block type	Tense	Tense	Voicing	Voicing	VOT	VOT
Deviant type	Present	Past	+voi	−voi	+asp	−asp
Critical deviants	pave	gave	ba	pa	pa	ba
	get	met	da	ta		
	thank	sank	ga	ka		
			va	fa		
			za	sa		
Critical standards	gave	pave	pa	ba	ba	pa
	met	get	ta	da		
	sank	thank	ka	ga		
			fa	va		
			sa	za		
Extra standards	chose	choose				
	sang	sing				
	bled	bleed				
	swore	swear				
	clung	cling				
	pled	plead				
	grew	grow				
	drew	woo				
	brought	bring				

*Note*. VOT = voice onset time; asp = aspirated.

In addition to these words, we added a set of “filler” standards for each block. We did this to avoid the possibility that participants could show an MMN just by tracking word frequencies. For example, in a block where present-tense verbs (*pave*, *get*, *thank*) are presented as deviants and past-tense verbs (*gave*, *met*, *sank*) as standards, the participants might notice that the latter words occur much more often in the block and that might be enough to elicit an MMN, without any role of the abstract linguistic differences between these two groups of words. Therefore, we added another nine present-tense irregular verbs to serve as extra standards, such that the block includes 12 standard tokens and three deviant tokens. (Most of the filler standards were past–present tense pairs of the same verb, but one pair—*drew-woo*—used two different verbs; we avoided present-tense “draw” because it can be a noun, e.g., *Team India played to a draw with Australia*.) Each token was repeated a similar number of times, so participants could not recognize an oddball arrangement simply by noticing how often certain words are repeated; the only way an oddball arrangement could be realized is if the brain notices that most of the stimuli in the block are past-tense verbs and only a few are present-tense verbs.

The phonological control blocks, on the other hand, include voiced or unvoiced consonants all embedded in the same [_**a**] context. One block used voiced consonants as deviants and unvoiced consonants as standards; the other block, vice versa. Thus, as in Monahan et al. (2022), there are no consistent acoustic cues to the voice difference. In order to keep this block as comparable as possible to previous research, we also did not add additional filler standards.

Examples of what a series of trials may look like within each block are shown in (1–4); deviants are indicated in bold.

1. **Tense contrast, past-tense deviant:** pave swear cling bleed grow **gave** choose get sue bring **sank** …
2. **Tense contrast, present-tense deviant:** gave swore clung bled grew **pave** chose met drew brought **thank** …
3. **Voicing contrast, voiced deviant:** pa ta ka pa ka **ba** fa sa ta sa **za** …
4. **Voicing contrast, voiceless deviant:** ba da ga ba ga **pa** va za da za **sa** …
5. **VOT contrast, aspirated deviant:** ba ba ba ba ba **pa** ba ba ba ba **pa** …
6. **VOT contrast, unaspirated deviant:** pa pa pa pa pa **ba** pa pa pa pa **ba** …

The words were read aloud by a male native English speaker from the United States. The speaker read five word lists where each contained the materials in a random order with fillers added as the first five and final five words of the list to ensure a more neutral tone and pace for the relevant words and to avoid fatigue effects. The audio recording is of studio quality: the words are recorded through a Studio Projects C3 microphone in an isolation booth with an Avid ICON D-Control ES 32 Fader w/ XMON mixing console acquired through Avid Pro Tools 10 software. Audio was sampled at 44.1 kHz with 32 bits per sample. After choosing a typical-sounding (with respect to tone and duration) recording for each word used in the experiment, the tokens were normalized to have the same average RMS intensity in Praat (Boersma & Weenink, 2021).

### Procedure

Participants read the participant information sheet and filled out a questionnaire on demographic details and the informed consent form. While the electroencephalography (EEG) cap was prepared, the experimenter explained to the participant that they will be watching a movie (the same nature documentary was provided for each participant), they will have multiple opportunities for breaks, they can blink normally but should avoid closing their eyes for an extended period of time, and they should attempt to sit still until a break.

The experiment proper was divided into 18 blocks. Each block of the experiment began with 16 standards, in order to get the participant habituated. Following that, for the rest of the block, trials were presented in a pseudorandom order such that a deviant was always preceded by 4–9 standards, and each block’s pseudorandom sequence contained 328 trials consisting of 40 deviants and 271 standards (this standard count does not include the 16 standards at the beginning of each block and 1 standard at the end of the block). Every time a deviant or standard was to be presented, the script randomly chose a token at runtime; this ensured that each of the three deviants and each of the 12 standards was chosen with similar frequency.

Each type of block was repeated three times, yielding a total of 18 blocks (5,904 trials) for one experiment session per participant; the order of the blocks is pseudorandomized such that the same condition did not occur for two blocks in a row. Between blocks, participants were able to take a self-paced break and were reminded of the current block number and the number of blocks remaining. Each block lasted approximately 8–9 min (variation due to the random inter-trial intervals (ITIs) and, for the past/present and voiced/voiceless blocks, trial-by-trial random selection of tokens from a set of tokens which have different durations), and depending on the time taken for between-block breaks, a whole experiment session lasted approximately 2 h and 45 min. The experiment was paused to check impedance after every six blocks.

While this is fairly long for an ERP experiment, we judged that any loss in data quality due to participant fatigue would be smaller than the losses in data quality that would be brought about by other alternatives such as lowering the number of trials (which would reduce power) or splitting the experiment across multiple sessions (which would introduce noise based on slight differences in electrode placement and would lead to participant attrition). We also note that, because participants get to watch movies during the experiment, 3 h of this experiment may be less fatiguing than 3 h of most other ERP experiments that involve explicit tasks. We have been able to observe MMNs in similarly long experiments before (Politzer-Ahles et al., 2016), and Luck (2014, chap. 8 suppl.) speculates that decreases in data quality beyond 45 min into an experiment may be modest.

The presentation of stimuli and recording of triggers to the acquisition program was conducted through Presentation (Neurobehavioral Systems, Inc.). The audio stimuli were presented binaurally using tube earphones (Etymotic Research) that provide a degree of sound attenuation. The ITI randomly varied from 900 to 1,100 ms (specifically, each trial’s ITI was randomly sampled from a uniform distribution of integers from 900 to 1,100).

### EEG Acquisition and Preprocessing

Continuous EEG was recorded from the scalp with a 64-channel Ag/AgCl electrode cap with a 10/20 system, using a SynAmps 2 amplifier (NeuroScan, Charlotte, NC, USA) with an online sampling rate of 1000 Hz. Recordings were made in DC mode with no high-pass filter, and with a 400 Hz low-pass filter. To monitor eye movements, four electrodes—a polygraphic electrode placed below the left eye and three electrodes affixed to the cap (one above the left eye and two located on each of the outer canthi)—were used to form two bipolar channels to monitor vertical and horizontal electrooculography (EOG). One channel located halfway between Cz and CPz was used as the reference during recording, and AFz served as the ground. Impedances were kept below 5 kΩ. A Stimtracker (Cedrus) interfaced between the experiment presentation software and the EEG acquisition software.

The EEG data were acquired through Curry 7 (Compumedics NeuroScan), and the EEG file were exported in .cnt format and analyzed using EEGLAB version 14.1.2 (Delorme & Makeig, 2004) for preprocessing and FieldTrip version 2020-03-20 (Oostenveld et al., 2011) for statistics. The data were visually inspected for bad channels, and up to three per participant were interpolated if necessary. The continuous data were then re-referenced to the average of both mastoids and segmented into epochs from 150 ms before and 750 ms after onset. (These values were chosen to ensure that the epochs could be as long as possible without overlapping with adjacent epochs, given the 500 ms ITI and the shortest audio stimulus in our materials.) The epochs were then demeaned (for each channel in each epoch, the mean of the data from the entire epoch was subtracted from each data point; per Groppe et al., 2011, this procedure may yield better independent component analysis (ICA) decompositions than baseline correcting based on the pre-stimulus interval) and then subjected to ICA using the runica() algorithm in EEGLAB (Makeig et al., 1997) to compose the data into as many independent components as there are channels (excluding the mastoids, EOGs, and any bad channels). The components were visually inspected to identify components corresponding to blinks and saccades, which were removed (no more than three components per participant were removed). Following removal of blink and saccade components, the data were baseline corrected again using a 100 ms pre-stimulus baseline, and then epochs with artifact were marked for removal based on an amplitude threshold (trials that exceed 75 *μ*V within 150 ms before to 600 ms after stimulus onset were removed). Finally, a 30 Hz low-pass filter (using the default settings of the EEGLAB function pop_eegfiltnew) was applied.

For each condition, the MMN was calculated by comparing the ERP elicited by tokens when they are presented as standards to the ERP elicited by those same tokens when they are presented as deviants. This analysis is sometimes referred to as identity MMN, or iMMN (Monahan et al., 2022; Pulvermüller et al., 2006). (Here, we use the term “iMMN” when referring to our own results and analysis, but use the term MMN when talking about the mismatch negativity phenomenon more generally.) For example, the iMMN for the past-tense deviant condition was the average of the ERPs for *gave*, *met*, and *sank* when they were deviants, minus the average of the ERPs for *gave*, *met*, and *sank* when they were standards. The first series of standards in a block, the first deviant in a block, and the first standard after each deviant were excluded from analysis, as were any trials marked for artifacts. Since each block included 40 deviants and each condition occurred in three blocks, this yielded 117, that is, (40 − 1) * 3, deviants per condition, minus any deviants trials rejected for containing artifacts.

### Statistical Analysis

Statistical analysis was conducted using cluster-based permutation tests (Maris & Oostenveld, 2007) over all scalp channels and the entire post-stimulus epoch. This analysis identifies clusters of spatiotemporally adjacent data points where the difference between two conditions is significant, and then uses a permutation test based on a cluster-level test statistic to evaluate the significance of the difference between conditions (see Maris & Oostenveld, 2007, for a proof of how this controls familywise error rate). In our analysis, the threshold for including a data point in a cluster was one-tailed *p* < 0.1 (we use a less strict threshold in order to make the test more sensitive to weak, sustained effects; a more strict threshold would make it more sensitive to strong focal effects) and at least two spatial neighbors that also meet the threshold (i.e., *minnbchan* = 2 in the Fieldtrip implementation of the cluster-based test). The permutation test used 1,000 iterations.

For the control contrasts (voicing and VOT), one permutation test each was performed. For the relatively abstract manipulation check (voiced/unvoiced) conditions, the test compared the voiced deviants to the corresponding voiced standards; likewise, for the more low-level VOT manipulation check, the unaspirated (phonologically voiced) deviants were compared to the corresponding unaspirated (phonologically voiced) standards. In other words, for each of these contrasts we only examined the iMMN elicited by voiced (unaspirated) deviants, and test whether that iMMN is significantly different from zero. The reason for only examining these and not the reversed contrasts (e.g., iMMNs elicited by voiceless deviants) is because phonologically voiced segments may be underspecified for the voice contrast in English, and MMNs tend to be weak when the standard is underspecified (e.g., Eulitz & Lahiri, 2004; Hestvik & Durvasula, 2016; this has been shown specifically for English voicing by Hestvik & Durvasula, 2016, and Schluter et al., 2017). Therefore, it was possible that there could be little to no iMMN in the block with voiced standards and unvoiced deviants, but observable iMMN in the block with unvoiced standards and voiced deviants. Collapsing across conditions could weaken the iMMN (since it could involve averaging a condition that elicits little to no iMMN with a condition that does elicit iMMN) and lead us to wrongly fail to detect an iMMN. Therefore, analyzing only the conditions with phonologically specified standards should yield the best chance of observing an iMMN in these contrasts—as long as (a) our assumption that voiceless standard/voiced deviant blocks elicit the biggest iMMN is correct and (b) the loss in power due to including fewer trials in the average is smaller than the loss in power would have been from including many trials that elicit little to no iMMN anyway. (Power is a function not just of sample size but also of effect size, and averaging together trials that do and do not elicit iMMN would cut the effect size in half.) The alpha level for each of these two permutation tests (one for voiced deviants vs. voiced standards in the abstract voice contrast blocks, and one for unaspirated deviants vs. unaspirated standards in the simple acoustic manipulation check blocks) was set to 0.05. For both of these contrasts, we report an alternative exploratory analysis—including both contrast directions—in the Supporting Information.

For the critical tense manipulation, we likewise conducted one test, comparing the ERP elicited by the present-tense deviant to that elicited by the present-tense standard. The reason for focusing on this comparison was that, if one assumes that present-tense verbs are actually lexically underspecified for tense whereas past-tense verbs are specified, then one might predict little to no iMMN in the block with present-tense standards and past-tense deviants, but an observable MMN in the block with past-tense standards and present-tense deviants; in that case, averaging across tenses would make us risk missing an iMMN that is actually present in one block. To our knowledge, this issue has not been discussed in the literature on underspecification and speech, most of which focuses on phonological underspecification (see, e.g., Lahiri & Reetz, 2010); however, there are other areas where underspecification and/or markedness in morphosyntactic features has been shown to impact processing, such as in L2 sentence processing (see, e.g., Alemán Bañón et al., 2021).

While analyzing just this comparison could mean losing some power (since we will be analyzing ERPs based on half as many trials as what we would have gotten if we collapsed across tenses), we judged it to be a smaller loss of power than the alternatives. Collapsing across tenses (i.e., averaging past-tense and present-tense deviants to get one deviant ERP, and averaging past-tense and present-tense standards to get one standard ERP) would give the most power *if* both conditions do elicit similar iMMNs, but if one condition yields little iMMN because of underspecification then this collapsing would have severely reduced power (because it would have cut the effect size in half). Likewise, trying both the analyses described above (one focusing on just past-tense deviants, and one collapsing across tense) would have also reduced power because it would have necessitated reducing the alpha level to account for the fact that we are performing two comparisons. Thus, focusing on just the present-tense deviants and standards probably yields the most power in this experiment. We also report a collapsed-across-tenses comparison for exploratory purposes in the Supporting Information, but we do not use it for making conclusions about whether or not our predictions were supported.

#### Predictions

There were several possible patterns of results that this experiment could have yielded. The conclusions that each of these patterns could license are described below.

- Significant iMMNs in all three manipulations (tense manipulation, abstract voice manipulation, and simple VOT manipulation). This is the only pattern that would allow us to conclude that the MMN can be elicited by purely linguistic contrasts.
- Significant iMMN in the abstract voice manipulation and simple VOT manipulation but not in the tense manipulation. This would license a conclusion that, as far as the available evidence shows, the MMN cannot be elicited by contrasts without some physical correlate (but it could be a physical correlate that is not consistent across other dimensions of the stimuli, as in Monahan et al., 2022, and Phillips, Pellathy, & Marantz, 2000).
- Significant iMMN in the simple VOT manipulation but in neither the tense manipulation nor the abstract voice manipulation. This would license a conclusion that, as far as the available evidence shows, the MMN cannot be elicited by purely linguistic contrasts, but only by reliable physical contrasts. This pattern would be inconsistent with studies such as Monahan et al. (2022) and Phillips, Pellathy, and Marantz (2000).
- No significant iMMN in any manipulation. This is not expected based on any previous literature and would only license a conclusion that our experiment setup was just not capable of eliciting MMNs at all.
- We did not expect any other pattern, and if any other pattern is observed we would not be able to make any theoretical conclusions from it, given that no other pattern is predicted by any of the accounts discussed thus far. We assume our three manipulations to be in a sort of entailment relationship: The tense manipulation is more abstract than the voicing manipulation and the voicing manipulation is more abstract than the VOT manipulation, and thus if the MMN can be elicited by one of these manipulations it should also be able to be elicited by any of the less abstract manipulations as well. Thus, a pattern of, e.g., iMMN for the tense manipulation and VOT manipulation, but not for the abstract voicing manipulation, would be difficult to explain.

## RESULTS

**Figure 1.**
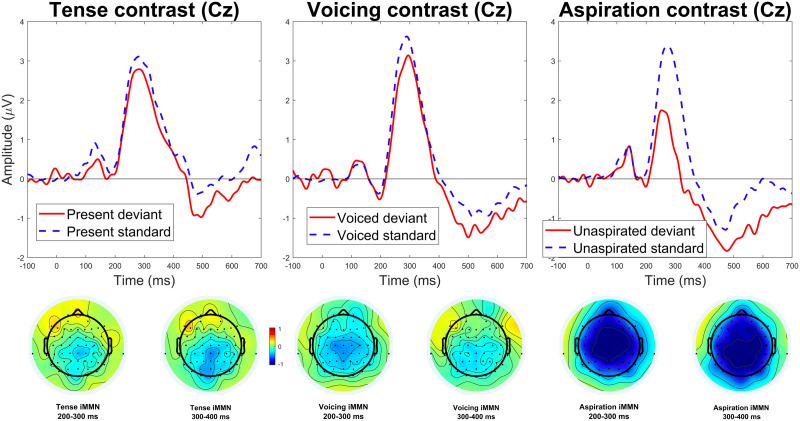
Event-related potentials for standards and deviants in each contrast, as well as topographic plots for the iMMN in each contrast in two time windows. Note, we use the term “identity mismatch negativity” (iMMN) when referring to our own results and analysis.

**Figure 2.**
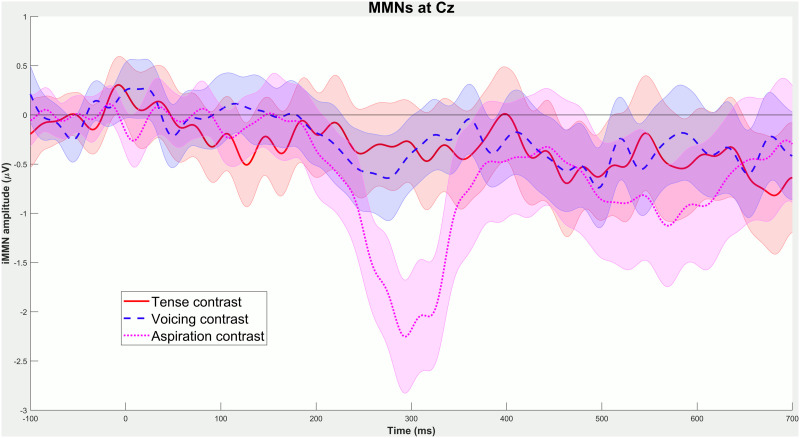
iMMN difference waves (at electrode Cz) for each contrast. Shaded ribbons indicate a 95% confidence interval of the mean event-related potential difference. The apparent pattern of significance suggested by this figure does not necessarily match the statistics reported in the text (e.g., the tense contrast, for which a significant iMMN is reported below, has a confidence interval that includes zero in this figure) because this figure is showing difference waves at a single electrode, whereas statistics were conducted using spatiotemporal cluster-based tests over the entire scalp. Where there are discrepancies between the figure and the text, the analysis reported in the text (which was pre-registered) takes precedence.

**Figure 3.**
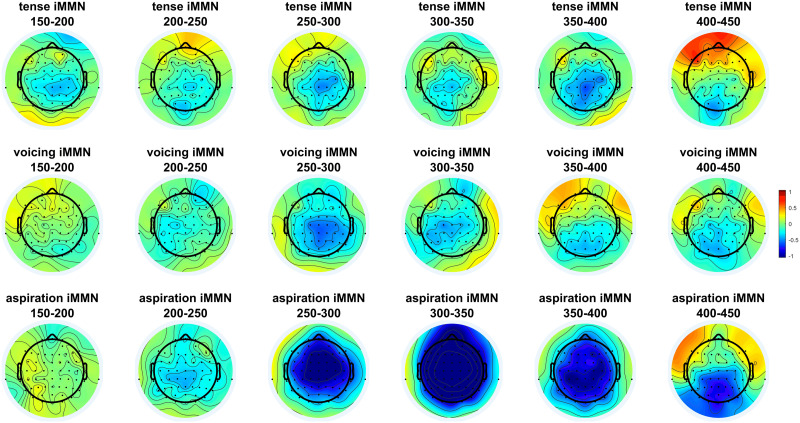
Topographic maps of the iMMN difference waves over several consecutive 50-ms time windows.

Figure 1 shows the ERPs elicited by standards and deviants in each contrast, and topographic maps of the iMMNs elicited by each contrast. Figure 2 compares the iMMN difference waves across different contrasts, and Figure 3 shows topographic maps of the deviant–standard differences over a range of time windows.

The contrast between aspirated and unaspirated stimuli clearly elicited a large iMMN effect, as it should. The other two contrasts were more subtle, but each appears to have elicited weak iMMN effects. The iMMN for the phonological voicing contrast was more centrally distributed rather than frontal—which is not inconsistent with the effect found by Monahan and colleagues (2022). The iMMN for the morphosyntactic tense contrast was even more posterior and emerged fairly late. All three contrasts also elicited negativities extending well beyond 400 ms.

Statistical analysis confirmed these observations. The difference between standards and deviants in the aspiration contrast was highly significant (*p* < 0.001), confirming that the experiment was capable of eliciting iMMNs. The difference between standards and deviants in the voicing contrast was also significant (*p* = 0.032), replicating Monahan et al. (2022) and indicating that the experiment was sensitive enough to detect the subtle iMMNs elicited by more abstract contrasts. Finally, and most importantly, the difference between standards and deviants in the morphosyntactic tense contrast was also significant (*p* = 0.037; this *p* value is based on a permutation test with 10,000 iterations, because the initial test with only 1,000 iterations yielded a borderline *p* value of 0.046).

### Exploratory Analysis

**Figure 4.**
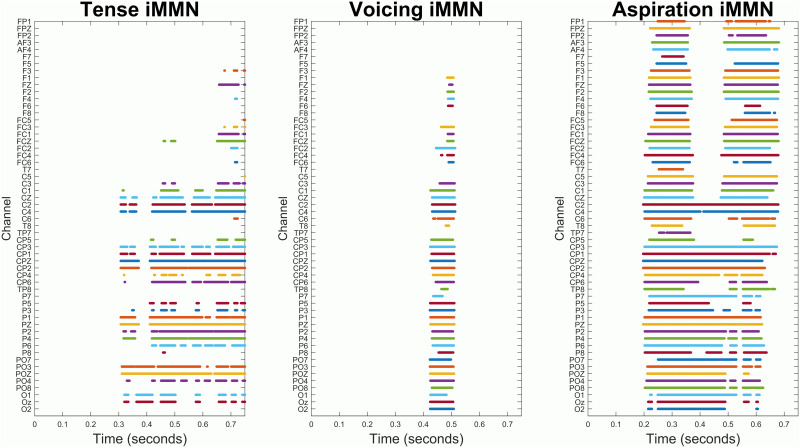
Raster plots showing which data points (which electrodes at which time points) were included in the cluster that was tested with the permutation test for each contrast. These graphs do not indicate that there were significant effects at these times or locations; rather, they are just a visual aid to understanding how the permutation test was conducted. These graphs can be interpreted as follows: When a contrast between two conditions was significant, that significant difference was driven by differences at the channels and time points shown in the graph. For example, the significant tense iMMN was driven mainly by differences between present-tense deviants and present-tense standards from 300 to 600 ms at centroposterior channels near the midline. The significant voicing iMMN, on the other hand, was driven by a broader group of channels, but over a narrower time window (about 400–600 ms).

While our pre-registered analysis found significant effects, inspection of the spatiotemporal distribution of the statistical clusters that drove these effects (Figure 4) reveals that these effects were driven mainly by differences in the late portion of the epoch, rather than in the MMN time window. In other words, the fact that present-tense deviants elicited significantly more negative ERPs than present-tense standards is not necessarily proof that they elicited an MMN; rather, they elicited a later negativity, and that is what drove this statistical effect. Furthermore, the figures above suggest that there are two separate standard–deviant differences, one before 400 ms and one after. Therefore, in order to test for an MMN more specifically, we ran a new exploratory analysis in which the statistical test was limited to the 100–400 ms time window, which is where we observed more MMN-like patterns. In this exploratory analysis, the aspiration and voicing contrasts again yielded significant effects (*p* = 0.001 and *p* = 0.013, respectively), but the critical tense contrast did not (*p* = 0.131).

These results are somewhat ambiguous, because while the pre-registered statistical analysis found a significant effect in the morphosyntactic tense contrast, follow-up analyses do not provide strong evidence that there is actually a significant iMMN within the time window typical for MMNs. While there is unambiguous evidence of a later negativity in this contrast, our analyses do not let us conclude that there was an early negativity. Visual inspection of the results suggests that there is a weak but consistent effect in roughly the MMN time window in centroposterior electrodes (e.g., Figure 3), but our statistical analyses were not able to capture it.

## DISCUSSION

The present pre-registered study tested whether a mismatch negativity can be elicited by an abstract linguistic contrast that is not cued by any reliable physical correlate: the distinction between [irregular] past- and present-tense English verbs. While many previous studies have shown that complex physical differences can elicit MMNs, and a small number of studies have shown that contrasts of two different linguistic categories without a single physical cue can also elicit MMNs, the present study is one of the first to examine whether the MMN detects differences in linguistic categories that do not use any reliable physical cues.

We found that a deviation in abstract linguistic category (a present-tense deviant among past-tense standards) indeed elicited a negativity, relative to the same stimulus presented in a context where it is not a deviant. Negativities were also elicited by phonological deviations (voiced deviants among voiceless standards) and by purely physical deviations (unaspirated deviants among aspirated standards). These results are consistent with our first prediction: significant iMMNs in all three contrasts (abstract tense contrast, abstract voicing contrast, and nonabstract aspiration contrast). Per our pre-registered analysis plan, this is the only possible pattern of results from our experiment that could have been consistent with the MMN being sensitive to purely linguistic abstract contrasts.

While the results provide evidence that the MMN may be elicited by an abstract contrast with no physical cue, there are some limitations that complicate this interpretation. Below we address (1) the question of whether the negativity elicited was indeed an MMN, and (2) the nature of the prediction that is involved in generating this negativity.

### Was This Effect an MMN?

The effect elicited by the tense contrast differed from the prototypical MMN in topography and timing. It was centroposterior rather than frontal, and it emerged much later than the MMN typically does. These differences raise the question of whether this was an MMN at all, or a modulation of some later component.

Centrally distributed negativities in MMN studies are not unheard of, particularly in MMN studies examining phonological contrasts. Hestvik and Durvasula (2016), Monahan and colleagues (2022), and Politzer-Ahles and colleagues (2016) all report fairly central MMNs. The MMN elicited by the phonological contrast in the present study appears consistent with these, and the effect elicited by the tense contrast appears similar or slightly more posterior, judging by visual inspection—we did not carry out direct statistical comparison of topographies. Therefore, we do not believe the topography of this effect rules out the possibility that it is an MMN.

The late emergence of this effect is also not out of the ordinary. For example, out of the studies mentioned above, Hestvik and Durvasula (2016) and Politzer-Ahles et al. (2016) also found effects that last into fairly late in the epoch, despite having early onsets. The effect in the present study is similar to those; while it is driven by differences late in the epoch, it begins to become visually apparent (albeit not statistically significant) early in the epoch. Furthermore, while we time-locked to stimulus onset for our ERP analysis, word disambiguation may have occurred later in the word, so the onset of this MMN may not have been very late relative to the actual moment at which the deviation is first detectable. Finally, difference detection that depends on higher-level processing of the stimuli may elicit later, and more posterior, negativities; see, for example, the theory articulated by Bornkessel-Schlesewsky and Schlesewsky (2019), according to which all language-related negativities (including MMNs) are based on the same predictive coding mechanism and only differ in terms of the nature of the prediction error that elicits them.

Finally, it is important to consider the nature of the task that elicited the present effect in light of the functional sensitivity of the MMN. The negativity in the present study was elicited in an inattentive oddball paradigm, in which participants’ attention was directed away from the auditory stimuli. This is a paradigm that is known to elicit MMN effects (as well as positive effects such as the P3b), whereas later language-related negativities such as the N400 are not typically elicited in such paradigms.

Based on these considerations, we tentatively conclude that the effect elicited by the tense contrast in this study was indeed an MMN, although we acknowledge that it has several differences from the canonical MMN which warrant further study. In particular, a source localization study would be valuable to provide a stronger test of whether this is truly an MMN. Further research into how inattentive this effect actually is—for example, whether it can be elicited with a distractor task, or while asleep, to ensure that participants really are not paying attention to the auditory stimuli—as well as research examining this contrast in both inattentive and attentive paradigms would also help clarify the functional sensitivity of this effect.

Our effect also bears similarity to the late discriminative negativity which is reported in some studies using oddball tasks, particularly in children (e.g., Čeponienė et al., 2002; Cheour et al., 2001; Martynova et al., 2003; Strotseva-Feinschmidt et al., 2015). This is a component that is elicited under similar circumstances as the MMN, but emerges later and with a more centroposterior distribution. It is mainly observed in children, and tends to be absent or greatly diminished in adults. The “MMN” observed in the tense and voicing contrasts in the present adult study is likewise late, centroposterior, and small. Further research would be needed to definitely determine whether this effect is an MMN or late discriminative negativity. Here we merely note that, given that the late discriminative negativity has the same functional characteristics as the MMN (specifically, they both can be elicited without attention directed toward the stimuli), the present results are still evidence for listeners’ pre-attentive sensitivity to abstract linguistic information.

### What Kind of Prediction Caused This Effect?

The negativity elicited by present-tense deviants amid past-tense standards was presumably based on some sort of prediction error—the word they encountered differed in some way from what they predicted. Current theories of the MMN (Bornkessel-Schlesewsky & Schlesewsky, 2019; Winkler, 2007) argue that the MMN is elicited when an incoming stimulus differs from a stimulus that was expected based on some rule-based regularity. In the classic oddball paradigm (such as the aspiration contrast used in the present study), where a participant hears sequences such as *ba ba ba ba ba ba pa*, the rule-based regularity is simple: A participant hearing a lot of *ba*s expects the next stimulus to be *ba*, and thus MMN is elicited when an incoming stimulus is instead *pa*. As reviewed in the Introduction, however, the rule-based regularities capable of eliciting MMN can be much more complex than this (see also Winkler, 2007, for review of complex regularities that have been shown to generate contexts where MMN effects can be elicited).

But what precisely were participants predicting? Below we consider two plausible possibilities.

First, participants may actually be predicting an abstract feature. Every time they hear a word, they extract a Past or Present feature from it (consistent with studies such as Fruchter et al., 2013, who show that irregular words are rapidly decomposed into abstract morphemes). Therefore, when they hear many past-tense standards, they begin to expect that feature. When they hear a present-tense deviant, they extract a Present feature, which mismatches with the Past feature they expected. In this case, the rule-based regularity—Past Past Past Past Past Past, leading the listener to expect the next word to also have a Past feature—is just as simple as the regularity in classic oddball paradigms such as our aspirated-unaspirated contrast. What makes this paradigm “abstract” is not the simple regularity itself, but the work involved in extracting the abstract tense feature from the stimuli in order to notice that regularity.

On the other hand, the findings of the present study could also be accounted for by assuming participants just predict a list of words. Recall that the present-tense standards consist of a list of 12 words that are repeated many times. With enough exposure to these words, participants may figure out that these words are the standards, so before each trial they are expecting to hear one of these 12 words. When they instead hear one of the deviants, it mismatches that expectation. This would be a less linguistically sophisticated form of processing than extracting abstract features as outlined above. Nevertheless, it would still be evidence for an MMN elicited by an abstract contrast, because it could only happen if the language processing system notices the past/present tense contrast. For this sort of prediction to happen, the parser would need to predict *only* the 12 past-tense standards and not the 3 present-tense deviants. Given that each of the words in a block (the 12 standards and the 3 deviants) is presented with the same frequency and there is no reliable physical difference between them, the only way for a participant to group the 12 standards and the 3 deviants into different categories is to notice that the 12 standards are all present-tense and the 3 deviants are all past-tense. In other words, while the nature of the prediction in this account is less abstract (predicting a list of words, rather than predicting an abstract feature), that prediction still cannot be made until an abstract feature has been detected by whatever system generates the predictive model. While it is not possible to rule out this account for the present study, it would be interesting to test a similar contrast in another language with a large number of irregular verbs (or with something else that would allow for an abstract contrast) so that the standards and deviants would not need to come from such a small set of words.

## CONCLUSION

In the present study, we observed an MMN elicited by an abstract linguistic contrast without any reliable physical cue: that between past-tense and present-tense irregular verbs. Specifically, irregular present tense verbs interspersed among irregular past tense verbs in a deviant–standard relationship elicited an MMN, even though there is no acoustic cue in the signal that reliably indicates the presence of past or present tense. These results provide the strongest evidence yet that the MMN is not merely a detector of physical differences, but rather is sensitive to differences in abstract linguistic categories.

## ACKNOWLEDGMENTS

We thank Kevin Schluter for assistance in conceptualizing this project. The article processing charges related to the publication of this article were supported by the University of Kansas (KU) One University Open Access Author Fund sponsored jointly by the KU Provost, KU Vice Chancellor for Research, and KUMC Vice Chancellor for Research and managed jointly by the Libraries at the Medical Center and KU–Lawrence.

## FUNDING INFORMATION

Stephen Politzer-Ahles, Hong Kong Research Grants Council General Research Fund, Award ID: 15605620.

## AUTHOR CONTRIBUTIONS

**Stephen Politzer-Ahles**: Conceptualization: Lead; Data curation: Equal; Formal analysis: Lead; Funding acquisition: Lead; Investigation: Supporting; Methodology: Lead; Project administration: Lead; Resources: Lead; Software: Lead; Supervision: Lead; Validation: Lead; Visualization: Lead; Writing – original draft: Lead; Writing – review & editing: Equal. **Bernard A. J. Jap**: Data curation: Equal; Formal analysis: Supporting; Investigation: Lead; Methodology: Supporting; Project administration: Supporting; Validation: Supporting; Visualization: Supporting; Writing – original draft: Supporting; Writing – review & editing: Equal.

## DATA AND CODE AVAILABILITY STATEMENT

Stimuli and experiment presentation code, anonymized data, and analysis code for this study are available at https://osf.io/yx9zm/.

## Supplementary Material


