# Supplementary material for: Can the Mismatch Negativity Really Be Elicited by Abstract Linguistic Contrasts?
Source: Neurobiol Lang (Camb). 2024 Sep 11;5(4):818–43. doi: 10.1162/nol_a_00147 (PMC11410353; doi:10.1162/nol_a_00147)
Supplement: Supplementary file 1 [file nol-5-4-818-s001.pdf]

Exploratory analysis with stricter inclusion criteria

Our originally pre-registered criteria for participant inclusion turned out to be very strict, so in the end we had relax the inclusion criteria (deviating from the original plan) in order to collect 60 participants' worth of usable data. Here we report an exploratory analysis of the data from only the 16 participants who met the original, strict inclusion criteria.

Figure S1 shows the ERPs elicited by standards and deviants in each contrast, and topographic maps of the MMNs elicited by each contrast. Figure S2 compares the MMN difference waves across different contrasts, and Figure S3 shows topographic maps of the deviant-standard differences over a range of time windows.

The difference between standards and deviants in the aspiration contrast was highly significant (p=.005), confirming that the experiment was capable of eliciting MMNs. However, there was no significant MMN for the voicing contrast (p=.399), nor for the tense contrast (no negative clusters detected in the test). Differences between the ERPs elicited by standards vs. by deviants in these conditions are apparent Figures S1 and S3, but did not reach statistical significance in this small sample.

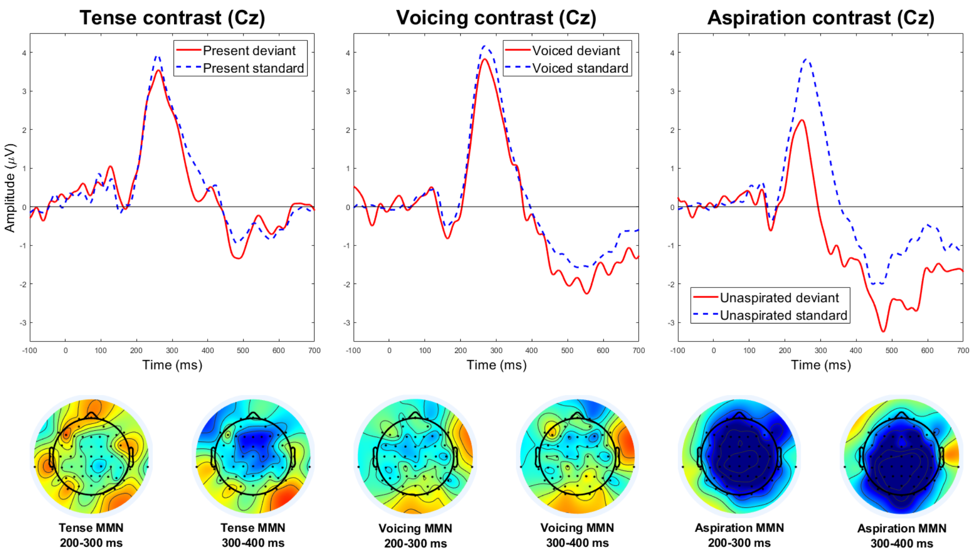

Figure S1. ERPs for standards and deviants in each contrast, as well as topographic plots for the MMN in each contrast in two time windows.

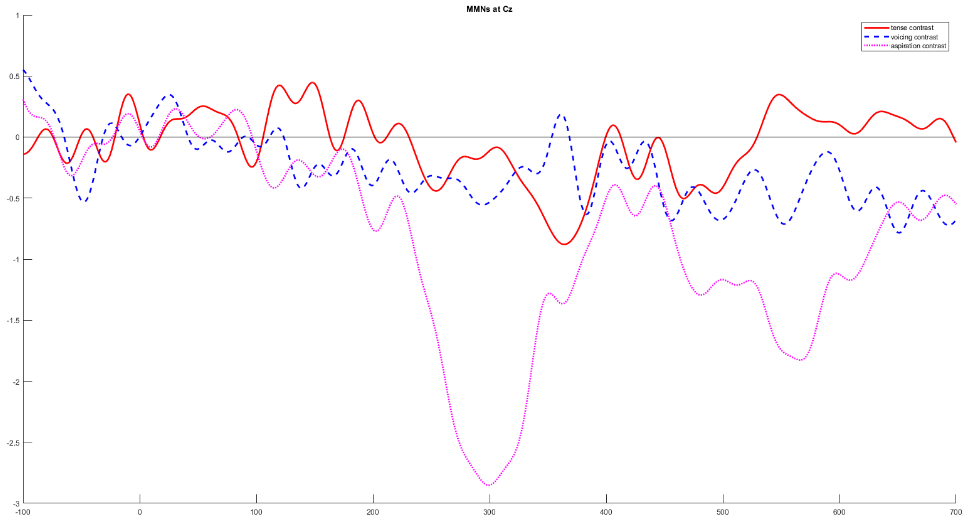

Figure S2. MMN difference waves (at electrode Cz) for each contrast.

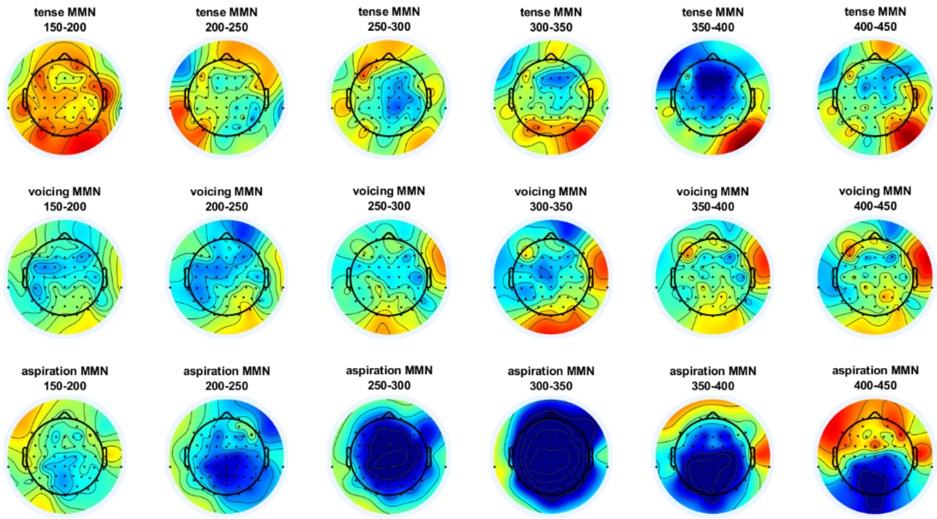

Figure S3. Topographic maps of the MMN difference waves over several consecutive 50-millisecond time windows.

Exploratory analysis collapsed across contrast direction

In our pre-registered analysis plan, we only analyzed ERPs elicited by unaspirated, voiced, or present-tense stimuli, since the other stimuli were not not expected to elicit MMNs. Here we report an additional analysis in which we analyzed the data from all stimuli (collapsing across aspirated and unaspirated, across voiced and voiceless, and across present- and past-tense). The aspirated-unaspirated contrast still yielded a highly significant MMN (p>.001), as did the voiced-voiceless contrast (p=.002). The tense contrast did not elicit a significant MMN (p=.503). These results are illustrated in Figures S4-S6.

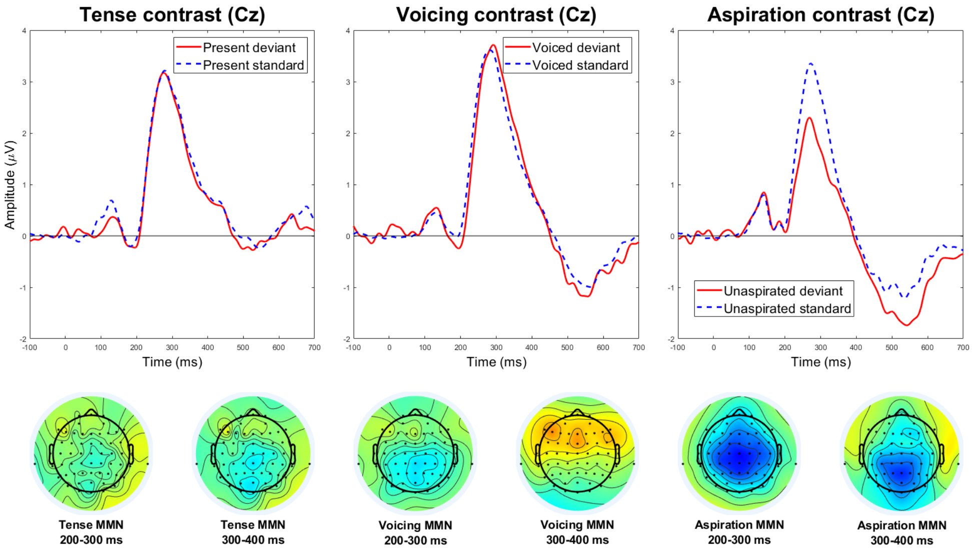

Figure S4. ERPs for standards and deviants in each contrast, as well as topographic plots for the MMN in each contrast in two time windows.

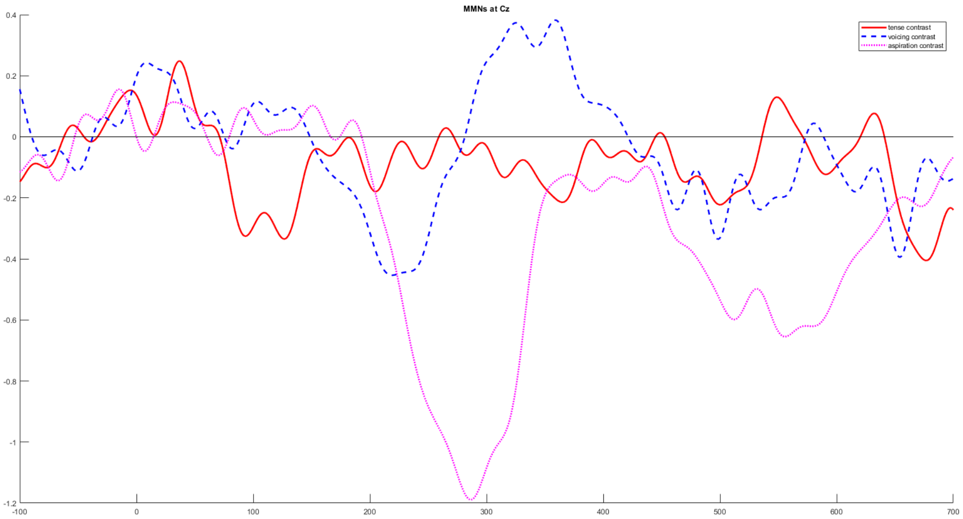

Figure S3. MMN difference waves (at electrode Cz) for each contrast.

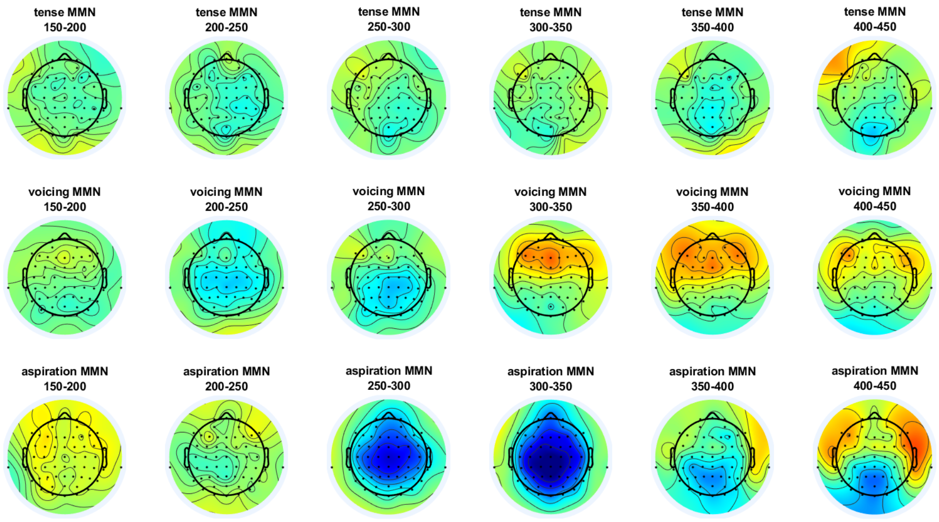

Figure S6. Topographic maps of the MMN difference waves over several consecutive 50-millisecond time windows.
